# Cannabis Use and Trajectories of Depression and Stress Across the Prenatal Period

**DOI:** 10.1001/jamanetworkopen.2024.51597

**Published:** 2024-12-17

**Authors:** Anna Constantino-Pettit, Rebecca Tillman, Jillian Wilson, Nicole Lashley-Simms, Naazanene Vatan, Azaria Atkinson, Shelby D. Leverett, Shannon Lenze, Christopher D. Smyser, Ryan Bogdan, Cynthia Rogers, Arpana Agrawal

**Affiliations:** 1 Department of Psychiatry, Washington University in St Louis, School of Medicine, St Louis, Missouri; 2 Washington University in St Louis School of Medicine, St Louis, Missouri; 3 Meharry Medical College School of Medicine, Nashville, Tennessee; 4 Division of Biology & Biomedical Sciences, Neurosciences Program, Washington University in St Louis, St Louis, Missouri; 5 Department of Neurology, Washington University in St Louis, St Louis, Missouri; 6 Department of Psychological and Brain Sciences, Washington University in St Louis, St Louis, Missouri

## Abstract

**Importance:**

Cannabis use among pregnant individuals has increased. Depression and stress are frequently reported motives for cannabis use that may prolong using cannabis during pregnancy.

**Objective:**

To examine associations between changes in depression, stress, and self-reported prenatal cannabis use (PCU), to examine motives for PCU, and to examine whether trajectories of depression and stress vary across individuals who report using cannabis to cope with mental health symptoms and/or stress, those who use cannabis for other reasons, and those who do not report PCU.

**Design, Setting, and Participants:**

This cohort study recruited pregnant individuals at an obstetric clinic at an academic hospital between July 2019 and January 2024 and followed them during pregnancy. Pregnant individuals with a history of lifetime cannabis use were included. Individuals reporting heavy episodic alcohol use or with other illicit drug use were excluded.

**Exposure:**

Self-reported PCU.

**Main Outcomes and Measures:**

The primary outcomes were self-reported depression (Edinburgh Postnatal Depression Scale), stress (Cohen Perceived Stress Scale), and cannabis use at each trimester during pregnancy, as well as motives for cannabis use during the first trimester (T1). Stability and changes in depression and stress scores and categorical self-reported prenatal cannabis use from T1 to the third trimester (T3) were estimated using individual linear growth curve models.

**Results:**

In this sample of 504 patients (all identified as women; median [IQR] age, 26 [18-40] years), 236 individuals (46.8%) reported PCU after pregnancy knowledge. Depression, stress, and PCU decreased from T1 to T3 (all slope estimates less than −0.29; SEs, 0.23-0.7; all *P *< .001). There were positive associations between depression and PCU at T1 (*r* = 0.17; *P* = .004) and in their rate of change (*r* = 0.18; *P* = .01). Only T1 stress and PCU were correlated (*r* = 0.14; *P* = .004). Participants reporting PCU for mental health reasons (137 participants [58.1%]) had the highest depression scores at each trimester; however, their rate of change in depression was statistically equivalent to those who did not use cannabis.

**Conclusions and Relevance:**

In this cohort study of PCU, participants who used cannabis did not experience a more significant decline in stress or depression symptoms compared with those who did not use cannabis. Individuals who used cannabis for mental health reasons did not hasten a decrease in their symptoms. Health care professionals are encouraged to enhance prenatal individuals’ access to empirically supported treatments for depression and stress.

## Key Points

**Question:**

Is prenatal cannabis use, particularly when used to reportedly cope with mood and stress, associated with changes in depression and stress across trimesters?

**Findings:**

In this cohort study of 504 patients, cannabis use during pregnancy was not associated with changes in depression and stress through pregnancy, even among those using cannabis to cope with mental health problems.

**Meaning:**

These findings suggest that cannabis use during pregnancy does not accelerate the rate of change in depression or stress, even when individuals report symptom relief as their motive for using the drug.

## Teaser

This cohort study examines associations between changes in depression, stress, and self-reported cannabis use during pregnancy.

## Introduction

Cannabis use during pregnancy has increased generationally.^1,2,3^ Although the American College of Obstetricians and Gynecologists discourages cannabis use during pregnancy and the preconception period, the proportion of birthing individuals who view cannabis as risky has decreased.^1^ Motives for using cannabis during the prenatal period vary; concerns related to nausea (eg, morning sickness) are predominant in the first trimester (T1) and early second trimester (T2) and, thus, may promote initial use.^4,5^ However, many individuals report cannabis use to cope with mental health conditions.^6,7,8,9^ This is particularly concerning as lifetime cannabis use has been robustly linked to increased likelihood of depression and anxiety, with studies suggesting that using a substance to cope with these psychiatric symptoms may index a more enduring pattern of use.^10^

Depression and stress are common during pregnancy, affecting 10% to 20% of pregnant individuals.^11,12,13,14^ As coping with mental health problems is a common motive for cannabis use, the increasing rates of depression and stress during pregnancy may promote cannabis use during pregnancy. However, as with many comorbid mood and substance use disorders, it is difficult to glean whether the mood disorder acts as the cause, the outcome, or some reciprocal agent in relation to substance use.^15,16,17^

These increasing rates raise questions regarding longitudinal trends in cannabis use during pregnancy. They further beg the question of the efficacy of cannabis (and plausibly, δ-9 tetrahydrocannabinol, or THC) to ameliorate depression and stress, especially among those who cite prenatal use primarily for these purposes. We examined trajectories of self-reported depression, stress, and cannabis use throughout pregnancy in a cohort of pregnant individuals, a subset of whom reported using cannabis during their pregnancy. We hypothesized that (1) depression, cannabis use, and stress would decrease throughout the prenatal period; and (2) individuals using cannabis to cope with mental health symptom relief during pregnancy would have higher baseline depression and stress scores but would not report greater decreases in depression and stress over time as a function of their cannabis use, compared with those who were either not using cannabis or using cannabis for reasons other than mental health symptom relief.

## Methods

### Sample

The ongoing longitudinal Cannabis Use During Early Life and Development (CUDDEL) study examines associations between prenatal cannabis use (PCU) and pregnancy and offspring (birth to 18 months) outcomes.^18^ For this cohort study, pregnant individuals at an obstetric clinic at an academic hospital were recruited between July 2019 and January 2024. Eligible individuals reported a lifetime history of cannabis use prior to their current pregnancy. Exclusion criteria included incarceration; age younger than 18 years; active psychosis, mania, or suicidal ideation; multifetal pregnancy; and use of assisted reproductive therapy. Self-reported heavy alcohol use or self-reported use of illicit or potentially teratogenic substances after knowledge of pregnancy, or urine positivity for such substances at any point during pregnancy, were also exclusion criteria. This study followed the Strengthening the Reporting of Observational Studies in Epidemiology (STROBE) reporting guidelines and was approved by the Washington University School of Medicine institutional review board. Participants provided written informed consent.

### Measures

#### Participant Race and Ethnicity

Race and ethnicity were assessed to characterize the sociodemographic variability of our sample. Participants self-reported their race (response options included American Indian or Alaska Native, Asian, Black or African American, Hawaiian or Pacific Islander, White, and other [other was the last option in the REDCap survey, with no further definitions]) and ethnicity (response options included Hispanic or Latinx, non-Hispanic or Latinx, and unknown) at T1. Participants could endorse multiple race options, and ethnicity was queried separately from race.

#### Prenatal Depression and Stress

At each trimester, prenatal depression was measured using the Edinburgh Postnatal Depression Scale (EPDS),^19^ with scores ranging from 0 to 28 and higher scores denoting greater depressive symptoms. Stress was measured using the Cohen Perceived Stress Scale (PSS),^20^ with scores ranging from 0 to 40 and higher scores denoting greater stress symptoms. Continuous, untransformed data were used in all primary analyses. Post hoc sensitivity analyses, used a dichotomous clinical cutoff for depression (EPDS score ≥13) (eTable 1 in Supplement 1).^21^

#### Prenatal Cannabis Use

PCU was assessed using participant self-report but was corroborated with urine drug screens at each trimester, which were moderately well correlated (*r* = 0.7).^18^ Only self-reported data were used in the current study since cannabis use motives were not evaluated in those who did not self-report T1 use (see eTable 2 in Supplement 1 for descriptive results that exclude any discrepancies between self-report and urine toxicology; results unchanged). At each trimester visit, participants were queried about how often they used cannabis using an item that was adapted from the Cannabis Use Disorders Identification Test–Revised,^22^ with response categories of never (0), monthly or less (1), 2 to 4 times a month (2), 2 to 3 times a week (3), or 4 or more times a week (4). These data were treated categorically for the growth models and as a binary variable for the presence or absence of cannabis use at T1 after knowledge of pregnancy for analyses where EPDS and PSS scores were stratified by prenatal cannabis exposure.

#### Coping and Motives for Use

During T1, individuals reporting cannabis use were asked, “Different women have different reasons for using marijuana during pregnancy. Please mark all that apply to you.” Responses included, “To deal with nausea/vomiting,” “To deal with anxiety or stress,” “To help with hunger or loss of appetite,” “To help you sleep,” “To treat mental health problems (e.g. depression, posttraumatic stress disorder),” “For enjoyment and relaxation,” “To have energy, get more things done during the day,” “To help with pain,” “It was uncomfortable to stop using,” and “Other.” Response options were derived from a variety of prior questionnaires, including the Pregnancy Risk Assessment Monitoring Survey.^23^ Individuals who marked either “To deal with anxiety or stress” or “To treat mental health problems (e.g. depression, posttraumatic stress disorder)” were characterized as using for mental health reasons. Those who reported using cannabis for other reasons were characterized as PCU for other reasons.

#### Income-to-Needs Ratios

Income-to-needs ratios (INRs) were derived by dividing total household income by the federal guideline for poverty, given the size of the family, in the year the participant responded to the questionnaire during T1. INRs ranged from 0.32 to 10.04, with lower INR denoting greater socioeconomic disadvantage.

#### History of Mental Health and Psychotropic Medication Use

A binary variable representing the presence of past or current mental health diagnosis was formed on the basis of participant electronic health records. The study protocol queried electronic health records only once, at delivery; thus, data were available for only a subset of participants who had already given birth. A binary variable was used to denote the presence of participant psychotropic medication prescriptions (ie, anxiolytics, antidepressants, stimulants, mood stabilizers, or antipsychotics prescribed to address mental health symptoms) ascertained from participant electronic health records extracted following childbirth.

#### Other Substance Use

Because heavy episodic alcohol use (by self-report) and use of other illicit substances (by self-report or urine toxicology) were exclusionary, rates of use were negligible.^18^ Thus, neither of these variables was used as covariates. Some of those who self-reported cannabis use at T1 (but who denied cigarette smoking or other nicotine use at screening) tested positive for cotinine, a nicotine metabolite, in their urine, potentially due to blunt usage or secondhand exposure in the past month. Sensitivity analyses (eTable 3 in Supplement 1) revealed no differences in EPDS or PSS scores when accounting for cotinine positivity as a covariate.

### Statistical Analysis

We estimated stability and change in EPDS (depression) and PSS (stress) scores and categorical self-reported PCU from T1 to T3 using individual linear growth curve models, covarying for age and INR. In a sensitivity analysis, we also included mental health history and psychotropic medication prescription as additional covariates. To examine the extent to which change in EPDS and PSS related to change in self-reported PCU, we extracted all intercepts and slopes and estimated correlations between them. Next, we included the extracted PCU intercept and slope as covariates in the EPDS and PSS linear growth models and regressed the EPDS and PSS slope and intercept on them. We also similarly regressed the PCU intercept and slope on extracted intercept-slope values for EPDS and PSS. Finally, to determine whether EPDS or PSS trajectories differed for individuals using cannabis to mitigate mental health symptoms, we fit a 3-group linear growth model for EPDS and PSS for participants who reported no PCU, those who reported PCU for mental health reasons, and those who reported PCU for other reasons. Intercepts and slopes were freely estimated in each of the 3 groups, and any nonsignificant estimates were constrained to zero for parsimony, followed by sequential equality constraints on the intercepts and slopes. Model fit was evaluated using the comparative fit index greater than 0.95 and root mean square error of approximation less than 0.06.^24^ Changes in model fit were calculated using the difference in −2 log likelihood for given degrees of freedom. Pairwise comparisons of EPDS and PSS means across groups were conducted using *t* tests, linear regressions, or generalized linear models in SAS statistical software version 9.4 (SAS Institute). All subsequent growth models were fitted to data in Mplus version 8 (Muthén and Muthén)^25^ with intercepts set at T1. Statistical significance was set at 2-tailed *P* < .05.

## Results

### Sample Characteristics

**Figure 1.**
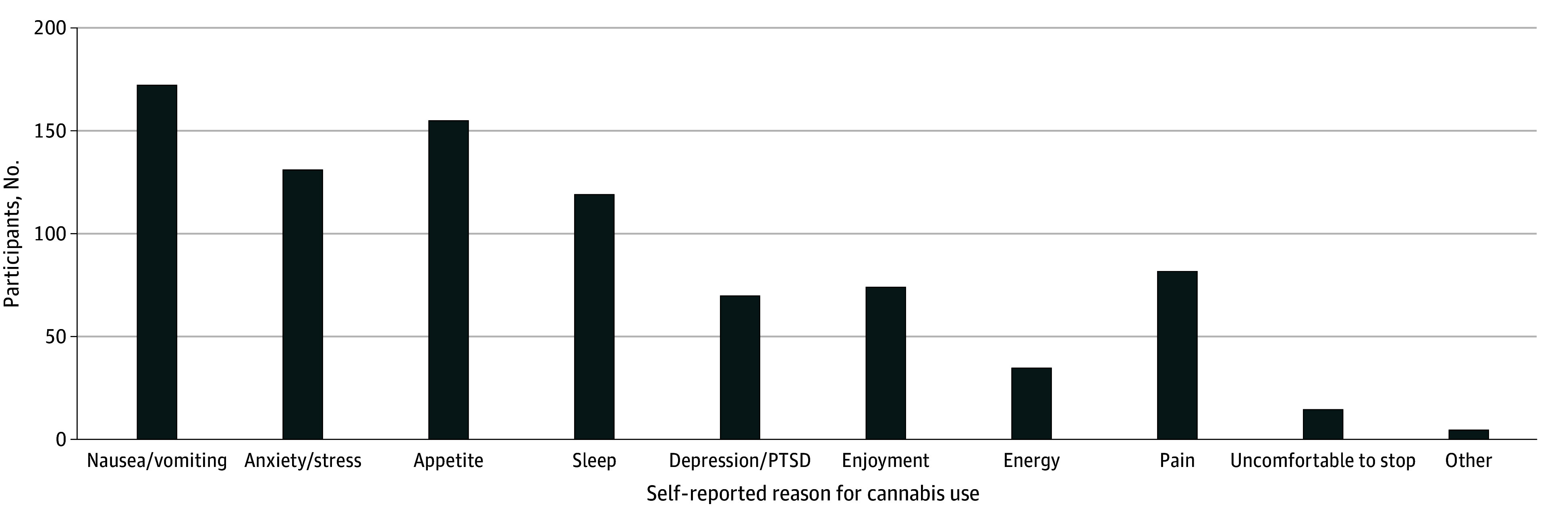
Frequency of Self-Reported Reasons for Cannabis Use During Pregnancy Among 236 Participants PTSD indicates posttraumatic stress disorder.

A total of 504 participants (all identified as women; median [IQR] age, 26 [18-40] years) were included. They had a mean (SE) INR of 1.20 (0.04) at T1. In total, 236 participants (46.8%) reported PCU, and 268 participants (53.2%) reported no PCU. Participants who reported PCU at T1 reported lower INR (ie, greater socioeconomic disadvantage), higher depression during T2, and higher stress during T3 (Table 1). T1 PCU (categorical) was not associated with data missingness at T2 (χ^2^_4_ = 6.45; *P* = .17) or T3 (χ^2^_4_ = 4.50; *P* = .34). There were no significant differences between the PCU and no PCU groups in terms of age, race, psychotropic medication use, or mental health history. Data for mental health history and use of psychotropic medications were available for only a subset of 428 participants. Only a few individuals had active prescriptions for psychotropic medications at their T1 visit (of these, 20 individuals reported PCU for mental health, 3 individuals reported PCU for other reasons, and 26 individuals reported no PCU), representing 25.8% of individuals with clinical depression (and 22.7% of those with prior mental health problems). Participants who reported PCU predominantly reported using cannabis to manage nausea and vomiting (172 participants [73%]), followed by managing hunger and appetite (155 participants [66%]) (Figure 1). Participants more frequently endorsed using cannabis for mental health symptom relief (137 participants [58.1%]) compared with other reasons, such as nausea, appetite, or sleep (99 participants [41.9%]). Of those who used cannabis at T1, participants who reported PCU for mental health reasons had more than twice the odds of continued use during T2 than those who reported PCU for other reasons (odds ratio, 2.77; 95% CI, 1.41-5.44; *P* = .003). Those who reported PCU for mental health had a greater likelihood of prior mental health diagnoses in their medical records (60.5%; 95% CI, 51.1%-69.3%) compared with the others those reporting no PCU (45.6%; 95% CI, 39.0%-52.3%) and those reporting PCU for other reasons (28.9%; 95% CI, 19.5%-39.9%).

**Table 1.** Sample Characteristics and Pairwise Comparisons Between Participants Who Reported PCU and Those Who Reported No PCU

Variable	Participants, No. (%)^a^			*P* value, no PCU vs PCU
	Total (N = 504)	No PCU (n = 268)	PCU (n = 236)	
Trimester 1 (mean, 14.7 wk)				
EPDS score, mean (SD) (n = 471)^b^	7.8 (5.8)^c^	7.4 (5.9)	8.2 (5.6)	.13
PSS score, mean (SD) (n = 414)^d^	16.9 (7.7)^c^	16.4 (8.0)	17.5 (7.3)	.16
PCU (n = 504)				
Never	268 (53.2)	NA	NA	NA
Monthly or less	53 (15.9)	NA	NA	NA
2-4 Times/mo	53 (10.5)	NA	NA	NA
2-3 Times/wk	65 (12.9)	NA	NA	NA
≥4 Times/wk	38 (7.5)	NA	NA	NA
Trimester 2 (mean, 25.1 wk)				
EPDS score, mean (SD) (n = 373)^b^	6.6 (5.7)^c^	5.9 (5.6)	7.4 (5.7)	.02
PSS score, mean (SD) (n = 320)^d^	15.7 (7.3)^c^	15.0 (7.6)	16.5 (6.8)	.06
PCU (n = 377)				
Never	223 (59.2)	NA	NA	NA
Monthly or less	49 (13.0)	NA	NA	NA
2-4 Times/mo	40 (10.6)	NA	NA	NA
2-3 Times/wk	39 (10.3)	NA	NA	NA
≥4 Times/wk	26 (6.9)	NA	NA	NA
Trimester 3 (mean, 34.2 wk)				
EPDS score, mean (SD) (n = 337)^b^	6.1 (5.4)^c^	5.8 (5.4)	6.6 (5.4)	.16
PSS score, mean (SD) (n = 284)^d^	15.1 (7.0)^c^	14.4 (7.2)	16.1 (6.6)	.04
PCU (n = 344)				
Never	225 (65.4)	NA	NA	NA
Monthly or less	41 (11.9)	NA	NA	NA
2-4 Times/mo	30 (8.7)	NA	NA	NA
2-4 Times/wk	26 (7.6)	NA	NA	NA
≥4 Times/wk	22 (6.4)	NA	NA	NA
Age, mean (SD), y	26.3 (4.9)	26.6 (5.1)	25.8 (4.7)	.08
Income-to-needs ratio, mean (SD)^e^	1.2 (1.0)	1.4 (1.2)	1.1 (0.6)	<.001
Self-reported race				
American Indian or Alaska Native	2 (<0.1)	2 (<0.1)	0	NA
Asian	2 (<0.1)	2 (<0.1)	0	NA
Black or African American	440 (87.1)	230 (85.8)	210 (88.9)	.35
Hawaiian or Pacific Islander	0	0	0	NA
White	54 (11.7)	33 (13.2)	21 (10.1)	NA
Other^f^	2 (<0.1)	2 (<0.1)	0	NA
Receipt of psychotropic medication in first trimester (n = 428)	49 (11.4)	26 (11.5)	23 (11.4)	1.0
Psychiatric illness (n = 428)	199 (46.5)	103 (45.6)	96 (47.5)	.76

Abbreviations: EPDS, Edinburgh Postnatal Depression Scale; NA, not applicable; PCU, prenatal cannabis use; PSS, Perceived Stress Scale.

^a^ Numbers of respondents at each trimester varied owing to the longitudinal design and ongoing nature of the study.

^b^ Scores for the EPDS range from 0 to 28, with higher scores denoting greater depressive symptoms.

^c^ Indicates significant post hoc pairwise comparisons after repeated measures analysis of variance for EPDS and PSS. Within trimesters, those who reported PCU but did not have EPDS or PSS data did not show statistically significant differences in PCU levels, self-reported race, age or income-to-needs ratio (all *P* > .10), with the exception of PSS at the third trimester, which was associated (*P* = .04) such that those without usable data for PSS were slightly more likely to report higher PCU engagement, although we note that third trimester data collection are ongoing; hence, this data point is not complete from a study perspective.

^d^ Scores for the PSS range from 0 to 40, with higher scores denoting greater stress symptoms.

^e^ Income-to-needs ratios were derived by dividing total household income by the federal guideline for poverty, given the size of the family, in the year the participant responded to the questionnaire during T1. They ranged from 0.32 to 10.04, with lower values denoting greater socioeconomic disadvantage.

^f^ Other was the last option in the REDCap survey, with no further definitions.

### Longitudinal Changes in Depression, Stress, and Cannabis Use

**Figure 2.**
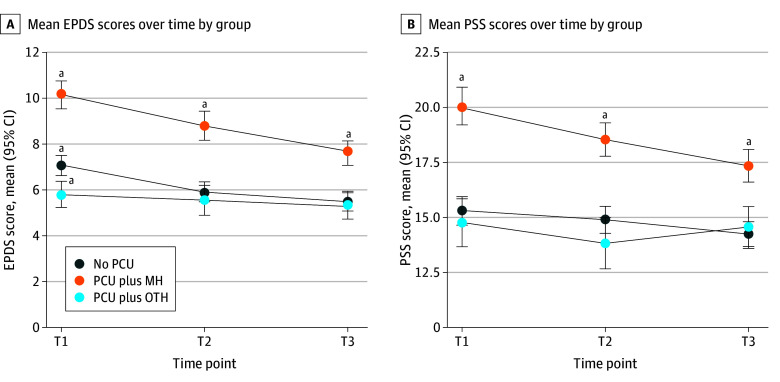
Measures of Depression and Stress Across Trimesters A, Depression was measured with the Edinburgh Postnatal Depression Scale (EPDS). B, Stress was measured with the Perceived Stress Scale (PSS). MH indicates mental health; OTH, other; PCU, prenatal cannabis use; T, trimester. ^a^Denotes significant differences in group means at the *P* < .05 level.

Depression, stress, and cannabis use declined across trimesters (Figure 2 and eFigure in Supplement 1). Intervariable correlations were high between depression and stress and modest with PCU (eTable 4 in Supplement). PCU at any trimester was unrelated to change in depression or stress (eTable 5 in Supplement 1). Depression and stress were only significantly lower in T2 relative to T1. Despite not being statistically significant, T3 depression and stress values were marginally lower than those for T2. Variance in depression and stress evidenced a slight decrease across trimesters as well. These descriptive analyses suggested reciprocal associations between PCU, depression, and stress at each trimester, but provided little support for an association between PCU and between-trimester change in depression or stress.

Unadjusted and covariate (age and INR) adjusted linear growth curve models for depression, stress, and self-reported PCU from T1 to T3 fit the data well (all slope estimates less than −0.29; SEs, 0.23-0.7; all *P *< .001) (Table 2). Age, but not INR, was negatively associated with T1 depression and stress. Conversely, INR but not age was negatively associated with T1 PCU. Covariates did not impact slope (ie, rate of change) estimates. The intercept-slope correlation was negative, suggesting that higher depression and stress at T1 were associated with a steeper decline into T3 (Table 2). T1 mental health history and psychotropic medication use were not associated with T1 PCU, although both were positively associated with depression slope and intercept, as well as stress slope (eTable 6 in Supplement 1). Because these additional covariates were unrelated to PCU, they were not included in subsequent analyses.

**Table 2.** Standardized Estimates for Outcomes of Interest^a^

Model	β (SE)		
	Prenatal cannabis use (N = 504)	Prenatal stress (n = 417)	Prenatal depression (n = 478)
Intercept	0.09 (0.7)^b^	2.59 (0.17)^c^	1.58 (0.11)^c^
Slope	−0.64 (0.19)^c^	−0.29 (0.10)^c^	−0.35 (0.08)^c^
Slope-intercept correlation	−0.08 (0.23)	−0.49 (0.10)^c^	−0.45 (0.10)^c^
Effect on intercept			
No. of participants	484	412	470
Age	−0.05 (0.06)	−0.16 (0.06)^d^	−0.13 (0.06)^e^
INR	−0.27 (0.08)^d^	−0.03 (0.06)	0.04 (0.06)
Effect on slope			
Age	−0.17 (0.21)	0.07 (0.08)	0.05 (0.07)
INR	0.15 (0.27)	−0.07 (0.07)	−0.05 (0.07)

Abbreviations: CFI, comparative fit index; INR, income-to-needs ratio; RMSEA, root mean square error of approximation.

^a^ For prenatal cannabis use, in the unadjusted model, CFI = 1.00 and RMSEA = 0.01; in the model adjusted for age and INR, CFI = 1.00 and RMSEA = 0.00. For prenatal depression, in the unadjusted model, CFI = 1.00 and RMSEA = 0.04; in the model adjusted for age and INR, CFI = 0.99 and RMSEA = 0.04. For prenatal stress, in the unadjusted model, CFI = 1.00 and RMSEA = 0.00; in the model adjusted for age and INR, CFI = 1.00 and RMSEA = 0.00.

^b^ Estimated mean for a categorical variable.

^c^ *P* < .001.

^d^ *P* < .01.

^e^ *P* < .05.

### Association of PCU With Depression and Stress Across Trimesters

Estimated PCU and depression intercepts were correlated, as was their rate of change (eTable 7 in Supplement 1). PCU intercept was associated with depression intercept (*r* = 0.17; *P* = .004). Estimated PCU slope was also associated with depression slope (*r* = 0.18; *P* = .01). However, the PCU intercept (ie, T1 PCU) was not associated with the decline in depression throughout pregnancy (ie, depression slope). Similarly, although stress and PCU intercepts were correlated (*r* = 0.14; *P* = .004), their slopes were not. Depression and stress influenced each other across pregnancy (intercepts and slopes were significantly associated) (eTable 7 in Supplement 1). In summary, PCU during T1 was not associated with the extent to which depression and stress scores changed during pregnancy (and vice versa).

### PCU for Mental Health Symptom Relief

Individuals who reported PCU for mental health reasons had significantly higher depression and stress scores at each trimester compared with those who reported PCU for other reasons and those who did not report PCU (Figure 1). There was no T1 to T3 change in depression or stress scores in the group reporting PCU for other reasons (slope constrained to 0) (Table 3). Despite reporting T1 PCU to cope with mental health symptoms, depression and stress rates of change (slopes) were equivalent in the no PCU and PCU for mental health groups. Therefore, T1 to T3 change in depression and stress was the same in those who reported PCU for mental health reasons as those who did not report PCU.

**Table 3.** Individual Linear Growth Models of Standardized Estimated EPDS (Depression) and PSS (Stress) Scores Across the Prenatal Period by PCU Status

Model	EPDS score, β (SE)			PSS score, β (SE)		
	No PCU (n = 253)	PCU for other reasons (n = 94)	PCU for mental health (n = 131)	No PCU (n = 223)	PCU for other reasons (n = 78)	PCU for mental health (n = 116)
Initial model						
Intercept	1.54 (0.16)^a^	1.18 (0.16)^a^	2.37 (0.38)^a^	2.50 (0.24)^a^	2.26 (0.31)^a^	3.36 (0.44)^a^
Slope	−0.36 (0.12)^b^	−0.05 (0.12)	−0.68 (0.32)^c^	−0.34 (0.14)^b^	−0.04 (0.13)	−0.37 (0.18)^c^
Slope-intercept correlation	−0.35 (0.14)^b^	−0.68 (0.11)^a^	−0.27 (0.29)	−0.41 (0.14)^b^	−0.50 (0.12)^a^	−0.69 (0.11)^a^
Best-fitting model						
Intercept	1.56 (0.15)^a^	1.63 (0.22)^a^	2.30 (0.37)^a^	2.51 (0.24)^a^	2.92 (0.22)^a^	3.34 (0.43)^a^
Slope	−0.43 (0.12)^a^^,^^d^	0.00^e^	−0.51 (0.23)^c^^,^^d^	−0.36 (0.13)^b^^,^^d^	0.00	−0.32 (0.12)^b^^,^^d^
Slope-intercept correlation	−0.36 (0.13)^b^	0.00^e^	−0.28 (0.28)	−0.41 (0.14)^b^	0.00	−0.69 (0.11)^a^

Abbreviations: EPDS, Edinburgh Postnatal Depression Scale; PCU, prenatal cannabis use; PSS, Perceived Stress Scale.

^a^ *P* < .001.

^b^ *P* < .01.

^c^ *P* < .05.

^d^ Estimates were statistically constrained to each other; slight differences are noted due to standardization of resulting estimate values.

^e^ Because there was no change in slope, it was constrained to 0.

## Discussion

This cohort study characterized trajectories of self-reported PCU, depression (EPDS), and stress (PSS) throughout pregnancy. Depression and stress during the T1 were individually and positively correlated with T1 cannabis use, consistent with other studies.^26,27^ Three novel findings emerged from the current study: (1) the decline in depression was associated with cannabis use decline, (2) cannabis use at T1 was not associated with changes in depression or stress over time, and (3) PCU to alleviate stress and mental health symptoms did not result in accelerated declines in either depression or stress, compared with not using cannabis during pregnancy. Those who used cannabis for other reasons reported lower and stable depression and stress scores throughout their pregnancy.

Because perinatal sleep difficulties and pain are known to influence mental health symptoms,^28,29^ we conducted multigroup analyses where PCU for mental health reasons was redefined to include endorsement of sleep difficulties and pain management. This did not alter results (eTable 8 in Supplement 1). Nausea, vomiting, and appetite regulation were the most common reasons for use among using for other reasons. However, individuals who reported PCU for mental health reasons had more than twice the odds of T2 cannabis use (odds ratio, 2.77; 95% CI, 1.41-5.44; *P* = .003) compared with those who reported PCU for other reasons who reported nausea, vomiting and appetite issues, highlighting the impact of mental health motives for use into pregnancy when other physiological symptoms might subside.

The higher EPDS scores among individuals reporting PCU for mental health at T1 identifies a subset of individuals who were more affected by prenatal depression. Those who reported PCU for mental health had a greater likelihood of prior mental health diagnoses in their medical records (60.5%; 95% CI, 51.1%-69.3%) compared with the others those reporting no PCU (45.6%; 95% CI, 39.0%-52.3%) and those reporting PCU for other reasons (28.9%; 95% CI, 19.5%-39.9%). There was a considerable decrease in depression in individuals reporting PCU for mental health, such that it approximated the starting level of depression in those reporting no PCU, is worth considering. It is possible that using cannabis during pregnancy did provide some relief to those using for mental health reasons, although it is difficult to know whether this was a true effect of cannabis, a placebo effect (an expectation of symptom relief leading to subjective report of alleviation), or some other unmeasured factor that may have also contributed to symptom reduction among these individuals.

Importantly, our findings indicate that offspring of those reporting PCU for mental health might have prolonged in utero cannabis exposure, which has been implicated in low birth weight and behavioral outcomes during childhood.^28,29^ Only a few individuals had active prescriptions for psychotropic medications at their T1 visit (20 individuals reporting PCU for mental health, 3 individuals reporting PCU for other reasons, and 26 individuals reporting no PCU), representing 25.8% of individuals with clinical depression (and 22.7% of those with prior mental health problems). This discrepancy may be indicative of structural inequities that continue to affect minoritized populations or those more greatly affected by poverty, both of which were overrepresented in our sample. There continue to be substantial disparities in access to perinatal mental health treatment,^30,31,32^ warranting interventions. For instance, in addition to the recommended universal screening for depression during pregnancy,^33,34,35^ practitioners are encouraged to ask their patients how they are coping with the stress and low mood that frequently accompany pregnancy. Increasing social and community support, along with providing resources for financial, housing, and other fundamental needs, are also forms of attending to the emotional health of pregnant individuals that are distinct from psychotherapy or medication, and have been found to improve perinatal mental health.^36,37,38,39^ Finally, coping with low mood and stress is a frequent motive for lifetime cannabis use,^40,41^ notably in women.^42,43^ Therefore, those who report PCU for mental health may have previously used cannabis for such symptom relief, establishing a pattern of self-medication^44^ that continued into their pregnancy. We did not collect data on motives for cannabis use prior to pregnancy. Additional data on postpartum depression and stress may shed light on the continued association between these variables. Of note, cannabis did not have a significant association with mental health symptoms above and beyond psychotropic medications (eTable 9 in Supplement 1), potentially indicating that timely treatment of depression in women contemplating pregnancy may be of manifold benefit, especially in women using cannabis.

### Strengths and Limitations

Strengths of the study include the prospective evaluation of depression, stress and cannabis use, exclusion of other substances, and elimination of confounders that differ between individuals who ever use cannabis and those who never use it. The study should be interpreted with some caveats. First, CUDDEL participants are frequently underrepresented in large national studies, despite the elevated rates of unmet prenatal and postpartum mental health needs in these demographic groups.^45^ The generalizability of these data to other populations should be considered. Second, medical records provided limited data on history of mental health diagnoses, as well as usage of prescription medication. Our study was not designed to examine prenatal depression specifically, and, thus, our ability to contrast depression changes as a function of prenatal use of cannabis vs depression medication is limited. Furthermore, we did not account for any individuals receiving psychotherapy during their pregnancy. Third, 3 measurement time points (T1-T3) limited our ability to test for nonlinear slopes. Although the distribution of means and variances suggests no serious violations of linearity, we have continued to collect EPDS, PSS, and PCU data during the postpartum period, which will allow for these alternative models in the future. Similarly, the numbers of individuals in groups stratified by use motives (eg, PCU for mental health vs PCU for other reasons) may have limited our statistical power to detect potential differences in slopes.

## Conclusions

In this cohort study of 504 pregnant individuals, we examined trajectories of depression, stress, and cannabis use from T1 to T3 and found that participants who used cannabis did not experience reduced stress or depression symptoms compared with nonusers. Many studies on cannabis use during pregnancy focus on the impact of this prenatal exposure on fetal growth and offspring outcomes.^46,47,48,49^ Greater emphasis is needed on maternal prenatal well-being. Beyond the heightened risk of prolonged in utero exposure to their offspring, our study urges attentiveness to cannabis use in individuals experiencing mental health symptoms and calls for increased access to empirically supported and effective alternatives to cannabis during pregnancy in individuals with prenatal depression, particularly in those who report coping as a motive for their use.

## Notes

Supplement 1. **eTable 1. **Percentage [95% Confidence Intervals] of Individuals with Depression Scores (EPDS) in the Putative Clinical Range (>=13) across: Individuals with No Self-Reported Cannabis Use (at T1); Individuals Using Cannabis to Cope with Mental Health Conditions; and Individuals Using Cannabis for Reasons Other than Mental Health **eTable 2.** Mean EPDS and PSS scores after exclusion for discordance in Self-Reported Cannabis Use and Urine Toxicology at T1 **eTable 3.** Association (Regression Coefficient, Standard Error, p-value) between EPDS, PSS, and Cotinine Positivity (at T1, T2, or T3) in PCU Individuals **eTable 4.** Bivariate Pearson Correlations across EPDS, PSS, and PCU **eTable 5.** Overall and group-stratified mean change in EPDS and PSS scores between T1 and T2, and between T2 and T3. Mean absolute value (to account for increase and decrease in scores) is also shown. Results from univariate regressions (Coefficient, SE) between PCU and Difference in EPDS and PSS Scores between T1-T2 and T2-T3 **eTable 6.** Baseline Model: Standardized Estimates and 95% Confidence Intervals For Cannabis, PSS, and EPDS Trajectories; Covariate-Adjusted Model: Standardized Estimates for Covariates in the Models **eTable 7.** Pearson Correlations between Estimated Intercept and Slopes for PCU, EPDS, and PSS, Computed Post-Estimation **eTable 8.** Individual Linear Growth Models of EPDS (Depression) and PSS (Stress) Scores across T1-T3 for: Individuals who Never Used Cannabis; Those who Used it, Either for Stress, Mental Health Symptoms, Sleep or Pain Management; and Those who Used Cannabis for Other Reasons **eTable 9.** Mean EPDS by Trimester among Individuals Using for Mental Health Conditions and Individuals with No Self-Reported Cannabis Use, Stratified by Psychotropic Medication Use **eFigure.** Sample individual observed data for PCU (Panel A), EPDS (Panel B), PSS (Panel C) across T1-T3

Supplement 2.
Data Sharing Statement
