# Supplementary material for: Cannabis Use and Trajectories of Depression and Stress Across the Prenatal Period
Source: JAMA Netw Open. 2024 Dec 17;7(12):e2451597. doi: 10.1001/jamanetworkopen.2024.51597 (PMC11653121; doi:10.1001/jamanetworkopen.2024.51597)
Supplement: Supplement 1. — eTable 1. Percentage [95% Confidence Intervals] of Individuals with Depression Scores (EPDS) in the Putative Clinical Range (>=13) across: Individuals with No Self-Reported Cannabis Use (at T1); Individuals Using Cannabis to Cope with Mental Health Conditions; and Individuals Using Cannabis for Reasons Other than Mental Health eTable 2. Mean EPDS and PSS scores after exclusion for discordance in Self-Reported Cannabis Use and Urine Toxicology at T1 eTable 3. Association (Regression Coefficient, Standard Error, p-value) between EPDS, PSS, and Cotinine Positivity (at T1, T2, or T3) in PCU Individuals eTable 4. Bivariate Pearson Correlations across EPDS, PSS, and PCU eTable 5. Overall and group-stratified mean change in EPDS and PSS scores between T1 and T2, and between T2 and T3. Mean absolute value (to account for increase and decrease in scores) is also shown. Results from univariate regressions (Coefficient, SE) between PCU and Difference in EPDS and PSS Scores between T1-T2 and T2-T3 eTable 6. Baseline Model: Standardized Estimates and 95% Confidence Intervals For Cannabis, PSS, and EPDS Trajectories; Covariate-Adjusted Model: Standardized Estimates for Covariates in the Models eTable 7. Pearson Correlations between Estimated Intercept and Slopes for PCU, EPDS, and PSS, Computed Post-Estimation eTable 8. Individual Linear Growth Models of EPDS (Depression) and PSS (Stress) Scores across T1-T3 for: Individuals who Never Used Cannabis; Those who Used it, Either for Stress, Mental Health Symptoms, Sleep or Pain Management; and Those who Used Cannabis for Other Reasons eTable 9. Mean EPDS by Trimester among Individuals Using for Mental Health Conditions and Individuals with No Self-Reported Cannabis Use, Stratified by Psychotropic Medication Use eFigure. Sample individual observed data for PCU (Panel A), EPDS (Panel B), PSS (Panel C) across T1-T3 [file jamanetwopen-e2451597-s001.pdf]

Supplemental Online Content

Constantino-Pettit A, Tillman R, Wilson J, et al. Cannabis use and trajectories of depression and stress across the prenatal period. *JAMA Netw Open*. 2024;7(12):e2451597. doi:10.1001/jamanetworkopen.2024.51597

eTable 1. Percentage [95% Confidence Intervals] of Individuals with Depression Scores (EPDS) in the Putative Clinical Range (>=13) across: Individuals with No Self-Reported Cannabis Use (at T1); Individuals Using Cannabis to Cope with Mental Health Conditions; and Individuals Using Cannabis for Reasons Other than Mental Health
eTable 2. Mean EPDS and PSS scores after exclusion for discordance in Self-Reported Cannabis Use and Urine Toxicology at T1
eTable 3. Association (Regression Coefficient, Standard Error, p-value) between EPDS, PSS, and Cotinine Positivity (at T1, T2, or T3) in PCU Individuals
eTable 4. Bivariate Pearson Correlations across EPDS, PSS, and PCU
eTable 5. Overall and group-stratified mean change in EPDS and PSS scores between T1 and T2, and between T2 and T3. Mean absolute value (to account for increase and decrease in scores) is also shown. Results from univariate regressions (Coefficient, SE) between PCU and Difference in EPDS and PSS Scores between T1-T2 and T2-T3
eTable 6. Baseline Model: Standardized Estimates and 95% Confidence Intervals For Cannabis, PSS, and EPDS Trajectories; Covariate-Adjusted Model: Standardized Estimates for Covariates in the Models
eTable 7. Pearson Correlations between Estimated Intercept and Slopes for PCU, EPDS, and PSS, Computed Post-Estimation
eTable 8. Individual Linear Growth Models of EPDS (Depression) and PSS (Stress) Scores across T1-T3 for: Individuals who Never Used Cannabis; Those who Used it, Either for Stress, Mental Health Symptoms, Sleep or Pain Management; and Those who Used Cannabis for Other Reasons
eTable 9. Mean EPDS by Trimester among Individuals Using for Mental Health Conditions and Individuals with No Self-Reported Cannabis Use, Stratified by Psychotropic Medication Use
eFigure. Sample individual observed data for PCU (Panel A), EPDS (Panel B), PSS (Panel C) across T1-T3

This supplemental material has been provided by the authors to give readers additional information about their work.

© 2024 Constantino-Pettit A et al. *JAMA Network Open.*

eTable 1. Percentage [95% Confidence Intervals] of Individuals with Depression Scores (EPDS) in the Putative Clinical Range (>=13) across: Individuals with No Self-Reported Cannabis Use (at T1); Individuals Using Cannabis to Cope with Mental Health Conditions; and Individuals Using Cannabis for Reasons Other than Mental Health

	T1 (%, 95 C.I.)	T2 (%, 95 C.I.)	T3 (%, 95 C.I.)
**No Self-Reported Cannabis Use (N=251)**	19.5 [14.8-25.0]	13.5 [8.7-19.1]	13.5 [8.5-18.5]
**Using Cannabis for Mental Health (N=128)**	34.4 [26.2-43.3]	25.5 [17.5 -34.9]	19.6 [12.0-29.2]
**Using Cannabis for Other Reasons (N=92)**	8.7 [3.8-16.4]	10.8 [4.8 - 20.2]	9.0 [3.4-18.5]

N reflects individuals with T1 EPDS scores.

© 2024 Constantino-Pettit A et al. *JAMA Network Open.*

<u>eTable 2</u>. Mean EPDS and PSS scores after exclusion for discordance in Self-Reported Cannabis Use and Urine Toxicology at T1

Of those who self-reported any T1 cannabis use (i.e., either for mental health or other reasons), 48 individuals in each of the 'mental health' and 'other reason' groups did not test positive for cannabis metabolites at T1. Likewise, 44 individuals who did not self-report any T1 cannabis use tested positive for cannabis metabolites at T1.

	T1 EPDS	T2 EPDS	T3 EPDS	T1 PSS	T2 PSS	T3 PSS
**Mean [SD]**						
**No PCU (N=253)**	7.9 [6.1]*	6.3 [5.8]	6.0 [5.4]	16.7 [8.1]	15.0 [7.9]	14.6 [7.2]
**Using Cannabis for Other Reasons (N=131)**	5.4 [4.3]	5.5 [5.2]	5.2 [4.9]	14.4 [7.6]	13.4 [7.7]	13.1 [6.9]
**Using Cannabis for Mental Health Reasons (N=253)**	10.5 [5.7]	8.4 [5.8]	7.4 [5.1]	19.5 [7.2]	18.3 [5.7]	18.0 [5.4]

*Mean T1 EPDS varied based on discordance between self-report and urine toxicology (p=0.003; 7.4 vs 7.9). No other estimates differed upon exclusion of data from discordant individuals. See footnote to Table 3 which documents that results from the multi-group model remained largely unchanged upon exclusion of data from discordant individuals.

© 2024 Constantino-Pettit A et al. *JAMA Network Open.*

eTable 3. Association (Regression Coefficient, Standard Error, p-value) between EPDS, PSS, and Cotinine Positivity (at T1, T2, or T3) in PCU Individuals

	T1 EPDS	T2 EPDS	T3 EPDS	T1 PSS	T2 PSS	T3 PSS
**Cotinine positivity**	0.52, 0.76, 0.49	0.24, 0.85, 0.78	0.34, 0.86, 0.70	0.61, 1.06, 0.57	-0.35, 1.12, 0.76	-0.85, 1.19, 0.48

© 2024 Constantino-Pettit A et al. *JAMA Network Open.*

eTable 4. Bivariate Pearson Correlations across EPDS, PSS, and PCU

	T1EPDS	T2EPDS	T3EPDS	T1PSS	T2PSS	T3PSS	T1PCU	T2PCU	T3PCU
**T1EPDS**	1								
**T2EPDS**	0.57*	1							
**T3EPDS**	0.44*	0.62*	1						
**T1PSS**	0.62*	0.46*	0.42*	1					
**T2PSS**	0.52*	0.63*	0.49*	0.59*	1				
**T3PSS**	0.50*	0.54*	0.64*	0.47*	0.62*	1			
**T1PCU**	0.11*	0.15*	0.11*	0.13*	0.12*	0.16*	1		
**T2PCU**	0.17*	0.17*	0.17*	0.21*	0.18*	0.21*	0.68*	1	
**T3PCU**	0.06	0.12*	0.18*	0.16*	0.09	0.10	0.61*	0.77*	1

*Significant at $p < 0.05$

© 2024 Constantino-Pettit A et al. *JAMA Network Open.*

<u>eTable 5</u>. Overall and group-stratified mean change in EPDS and PSS scores between T1 and T2, and between T2 and T3. Mean absolute value (to account for increase and decrease in scores) is also shown. Results from univariate regressions (Coefficient, SE) between PCU and Difference in EPDS and PSS Scores between T1-T2 and T2-T3.

	Overall	T1 PCU	T2 PCU	T3 PCU
**T1-T2 difference in EPDS (Mean, SD)**<br>**Absolute value (Mean, SD)**	1.06 (5.3)<br>3.88 (3.8)	-	-	-
**T2-T3 difference in EPDS (Mean, SD)**<br>**Absolute value (Mean, SD)**	0.50 (4.9)<br>3.43 (3.5)	-	-	-
**T1-T2 EPDS**	-	-0.09, 0.20	-0.02, 0.21	-0.26, 0.23
**T2-T3 EPDS**	-	0.09, 0.20	0.04, 0.21	-0.18, 0.22
**T1-T2 difference in PSS (Mean, SD)**<br>**Absolute value (Mean, SD)**	0.94 (6.8)<br>5.14 (4.6)	-	-	-
**T2-T3 difference in PSS (Mean, SD)**<br>**Absolute value (Mean, SD)**	0.60 (6.2)<br>4.55 (4.30)	-	-	-
**T1-T2 PSS**	-	-0.17, 0.29	0.23, 0.29	0.50, 0.33
**T2-T3 PSS**	-	0.44, 0.29	-0.20, 0.30	-0.08, 0.33

None of the regression-based estimates are statistically significant at $p < 0.05$; results were unchanged when regressions were conducted using absolute value of difference in EPDS and PSS scores

© 2024 Constantino-Pettit A et al. *JAMA Network Open.*

eTable 6. Baseline Model: Standardized Estimates and 95% Confidence Intervals For Cannabis, PSS, and EPDS Trajectories; Covariate-Adjusted Model: Standardized Estimates for Covariates in the Models

Baseline Model	Prenatal Cannabis Use (*n=504*)			Prenatal Stress (*n=417*)			Prenatal Depression (*n=478*)		
	Intercept [95% CI]	Slope [95% CI]	Slope-Intercept Correlation [95% CI]	Intercept [95% CI]	Slope [95% CI]	Slope-Intercept Correlation [95% CI]	Intercept [95% CI]	Slope [95% CI]	Slope-Intercept Correlation [95% CI]
No covariates	0.90[&] [0.03-0.15]	-0.64 [-.95, -.34]	-0.08 [-.30, 0.46]	2.59 [2.31, 2.87]	-0.29 [-.43, -.15]	-0.49 [-.62, -.36]	1.58 [1.40, 1.77]	-0.35 [-.49, -.21]	-0.45 [-.58, -.31]
**Covariate-Adjusted Model**	**Prenatal Cannabis Use** (*n=411*)			**Prenatal Stress** (*n=372*)			**Prenatal Depression** (*n=397*)		
	Effect on Intercept	Effect on Slope	-	Effect on Intercept	Effect on Slope	-	Effect on Intercept	Effect on Slope	-
Age	-0.12	0.03	-	-0.15**	0.07	-	-0.12*	0.03	-
INR	-0.25**	0.22	-	-0.02	-0.08	-	0.03	-0.03	-
Mental Health History	0.02	0.01	-	0.23***	0.01	-	0.33***	-0.17*	-
Psychotropic Meds	0.04	-0.02	-	0.19**	-0.11	-	0.19**	-0.11	-

Significance levels: *.05, **.01, ***.001; [&]Estimated intercept and confidence intervals, not mean of raw data; Prenatal Depression (EPDS) model fit: Unadjusted (CFI = 1.00; RMSEA = 0.04); Adjusted for age, INR, mental health, and psychotropic medication (CFI = 0.99; RMSEA = 0.04); Prenatal Stress (PSS) model fit: Unadjusted (CFI = 1.00; RMSEA = 0.00); Adjusted for age, INR, mental health, and psychotropic medication (CFI = 1.00; RMSEA = 0.00); Prenatal Cannabis (PCU) model fit: Unadjusted (CFI = 1.00; RMSEA = 0.00); Adjusted for age, INR, mental health and psychotropic medications (CFI = 1.00; RMSEA = 0.00)

© 2024 Constantino-Pettit A et al. *JAMA Network Open.*

eTable 7. Pearson Correlations between Estimated Intercept and Slopes for PCU, EPDS, and PSS, Computed Post-Estimation.

For these estimates, intercepts and slopes were saved out from the linear growth models. These extracted estimates were then correlated, post-hoc.

	PCU Intercept	PCU Slope	EPDS Intercept	EPDS Slope	PSS Intercept	PSS Slope
**PCU Intercept**	1					
**PCU Slope**	0.23***	1				
**EPDS Intercept**	0.13**	0.03	1			
**EPDS Slope**	0.01	0.13**	-0.39***	1		
**PSS Intercept**	0.14**	0.05	0.70***	-0.18**	1	
**PSS Slope**	-0.01	-0.01	-0.20***	0.35***	-0.48***	1

**p<0.01 ***p<0.001

© 2024 Constantino-Pettit A et al. *JAMA Network Open.*

eTable 8. Individual Linear Growth Models of EPDS (Depression) and PSS (Stress) Scores across T1-T3 for: Individuals who Never Used Cannabis; Those who Used it, Either for Stress, Mental Health Symptoms, Sleep or Pain Management; and Those who Used Cannabis for Other Reasons

	EPDS (Depression)			***PSS (Stress)***		
	**Never Used Cannabis** *(n=253)*	**PCU-other motives** *(n=51)*	**PCU for stress, mental health, sleep or pain** *(n=174)*	**Never Used Cannabis** *(n=223)*	**PCU-other motives** *(n=44)*	**PCU for stress, mental health, sleep or pain** *(n=150)*
**Initial Model**						
Intercept	1.54 (0.16)***	1.38(0.30)***	1.84 (0.21)***	2.50 (0.24)***	2.62 (0.55)***	2.90 (0.31)***
Slope	-0.36 (0.12)**	-0.14 (0.17)	-0.41 (0.15)**	-0.34 (0.14)*	0.07 (0.19)	-0.33 (0.14)*
Slope-Intercept Correlation	-0.35 (0.14)**	-0.41 (0.02)*	-0.55 (0.13)***	-0.41 (0.14)**	-0.48 (0.19)**	-0.61 (0.10)***
**Best-fitting Model**						
Intercept	1.55 (0.15)***	1.72 (0.37)***	1.83 (0.21)***	2.51 (0.24)***	3.59 (0.78)***	2.89(0.30)***
Slope	-0.38(0.11)***[a]	0.00[&]	-0.37 (0.12)**[a]	-0.36(0.13)**[b]	0.00	-0.30 (0.10)**[b]
Slope-Intercept Correlation	-0.35 (0.13)**	0.00[&]	-0.55 (0.13)***	-0.41(0.14)**	0.00	-0.61 (0.10)***

Note: All estimates are standardized. Significance levels: *.05, **.01, ***.001. [&]Because there was no change in slope, it was constrained to 0; [a, b] Estimates with the same alphabetical superscript were equated; estimates differ slightly due to standardization

© 2024 Constantino-Pettit A et al. *JAMA Network Open.*

eTable 9. Mean EPDS by Trimester among Individuals Using for Mental Health Conditions and Individuals with No Self-Reported Cannabis Use, Stratified by Psychotropic Medication Use *

	T1	T2	T3
**Using for Mental Health with T1 medications [n=20]**	10.9 [8.3-13.6][ab]	7.5 [4.7-10.4][ab]	7.9 [4.9 - 10.8][abc]
**Using for Mental Health without T1 medications [n=98]**	9.8 [8.6-11.1][b]	8.6 [7.3-9.9][bc]	7.4 [6.1-8.7][a]
**Never-Users with T1 medication [n=26]**	12.5 [9.7-15.2][a]	9.5 [6.2-12.9][ac]	8.1[4.8-11.3][b]
**Never-Users without T1 medication [n=201]**	6.6 [5.9-7.4]	5.4 [4.6-6.2]	5.4 [4.6-6.3][c]

*Individuals using for other reasons not shown as number endorsing psychotropic medications was too low (n=3); [abc] estimates with the same alphabetical superscript were statistically equivalent to each other. N reflects those with valid EPDS data;

© 2024 Constantino-Pettit A et al. *JAMA Network Open.*

<u>eFigure</u>. Sample individual observed data for PCU (Panel A), EPDS (Panel B), PSS (Panel C) across T1-T3. *Plotted using the "plot" option in Mplus, within each linear growth analysis. Observed values are shown.*

Panel A: PCU

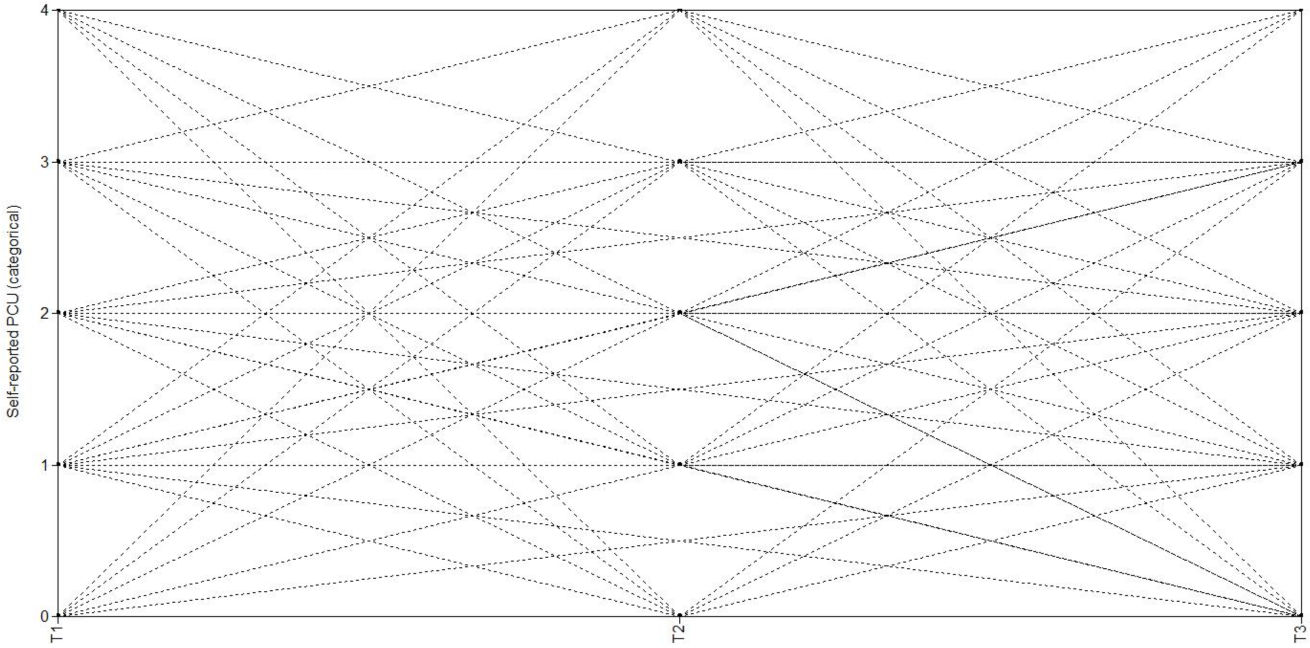

Panel B: EPDS

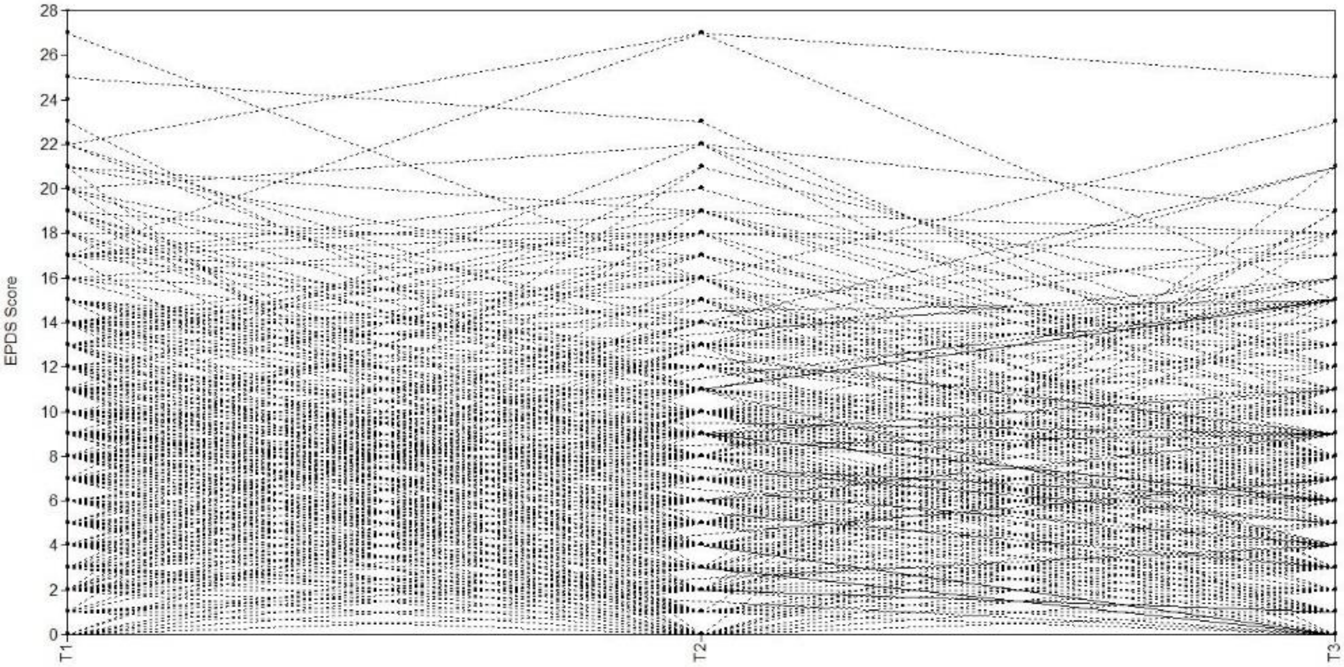

© 2024 Constantino-Pettit A et al. *JAMA Network Open.*

Panel C: PSS

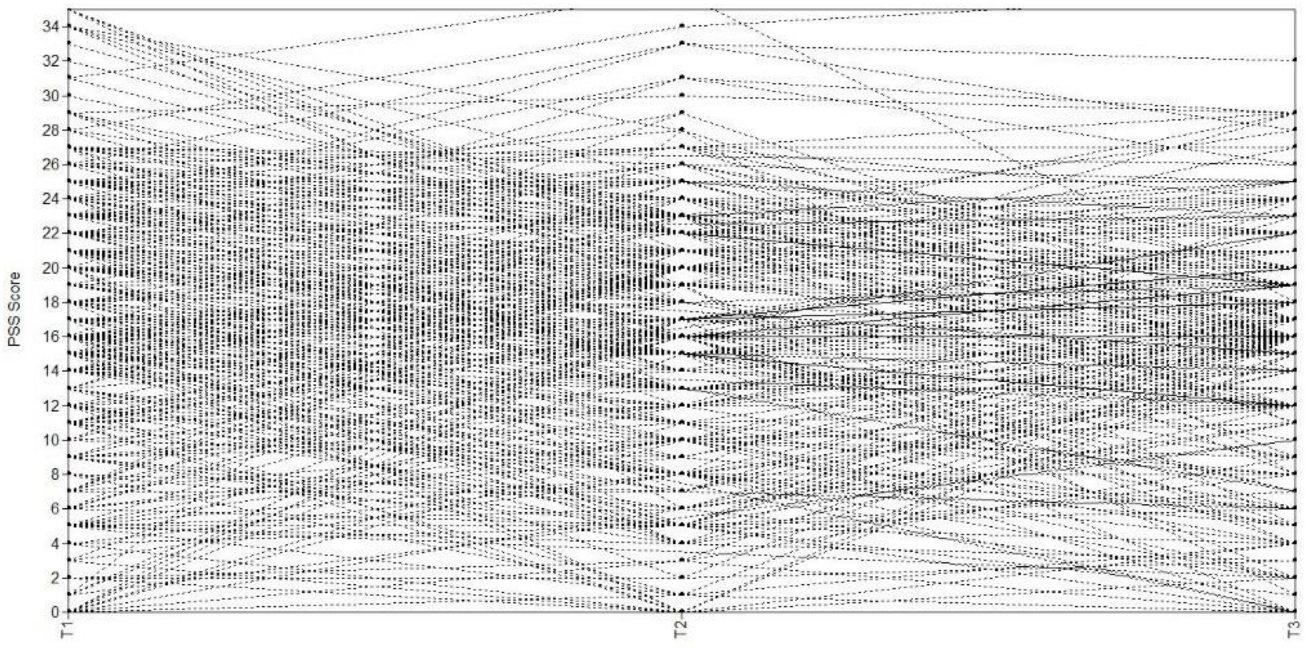

© 2024 Constantino-Pettit A et al. *JAMA Network Open.*
