# Supplementary material for: Cannabis Use and Trajectories of Depression and Stress Across the Prenatal Period
Source: JAMA Netw Open. 2024 Dec 17;7(12):e2451597. doi: 10.1001/jamanetworkopen.2024.51597 (PMC11653121; doi:10.1001/jamanetworkopen.2024.51597)
Supplement: Supplement 2. — Data Sharing Statement [file jamanetwopen-e2451597-s002.pdf]

Data Sharing Statement

Constantino-Pettit. Cannabis Use and Trajectories of Depression and Stress Across the Prenatal Period. *JAMA Netw Open*. Published December 17, 2024. doi:10.1001/jamanetworkopen.2024.51597

Data
Data available: No
